# EGFR controls bone development by negatively regulating mTOR-signaling during osteoblast differentiation

**DOI:** 10.1038/s41418-017-0054-7

**Published:** 2018-02-14

**Authors:** Markus Linder, Manfred Hecking, Elisabeth Glitzner, Karin Zwerina, Martin Holcmann, Latifa Bakiri, Maria Grazia Ruocco, Jan Tuckermann, Georg Schett, Erwin F. Wagner, Maria Sibilia

**Affiliations:** 1 Department of Internal Medicine I, Institute of Cancer Research, Comprehensive Cancer Center, Medical University of Vienna, Vienna, Austria; 2 Department of Internal Medicine 3, Friedrich-Alexander-University Erlangen-Nürnberg (FAU), Universitätsklinikum Erlangen, Erlangen, Germany; 3 Department of Internal Medicine III, Medical University of Vienna, Vienna, Austria; 4 Spanish National Cancer Research Center (CNIO), Madrid, Spain; 5 Institute of Comparative Molecular Endocrinology, University of Ulm, Ulm, Germany; 6 Present Address: Department of Internal Medicine III, Medical University of Vienna, Waehringer Guertel 18-20, Vienna, A-1090 Austria

## Abstract

Mice deficient in epidermal growth factor receptor (*Egfr*^−/−^ mice) are growth retarded and exhibit severe bone defects that are poorly understood. Here we show that EGFR-deficient mice are osteopenic and display impaired endochondral and intramembranous ossification resulting in irregular mineralization of their bones. This phenotype is recapitulated in mice lacking EGFR exclusively in osteoblasts, but not in mice lacking EGFR in osteoclasts indicating that osteoblasts are responsible for the bone phenotype. Experiments are presented demonstrating that signaling via EGFR stimulates osteoblast proliferation and inhibits their differentiation by suppression of the IGF-1R/mTOR-pathway via ERK1/2-dependent up-regulation of IGFBP-3. Osteoblasts from *Egfr*^−/−^ mice show increased levels of IGF-1R and hyperactivation of mTOR-pathway proteins, including enhanced phosphorylation of 4E-BP1 and S6. The same changes are also seen in *Egfr*^−/−^ bones. Importantly, pharmacological inhibition of mTOR with rapamycin decreases osteoblasts differentiation as well as rescues the low bone mass phenotype of *Egfr*^−/−^ fetuses. Our results demonstrate that suppression of the IGF-1R/mTOR-pathway by EGFR/ERK/IGFBP-3 signaling is necessary for balanced osteoblast maturation providing a mechanism for the skeletal phenotype observed in EGFR-deficient mice.

## Introduction

Skeletal development requires complex and coordinated interplay between mesenchymal cells—chondrocytes and osteoblasts—at various stages of differentiation. The succession of events whereby osteoblast formation follows chondrocyte differentiation resulting in bone formation in long bones of vertebras is termed “endochondral ossification” [1]. Differentiation of osteoblasts is induced by up-regulation of specific transcription factors accompanied by the expression of factors that facilitate mineralized extracellular matrix formation [2].

Genetic ablation of the epidermal growth factor receptor (EGFR) in mice revealed its intricate role during embryonic and postnatal development [3, 4]. Mice lacking the EGFR (*Egfr*^−/−^) have major organ defects and die either in utero or shortly after birth, depending on the genetic background [3-5]. Among other developmental abnormalities EGFR-deficient mice are severely growth retarded and exhibit skeletal defects [6]. We have previously reported that long bones of EGFR-deficient mice display a greatly increased zone of hypertrophic chondrocytes, suggesting that EGFR negatively regulates condrocyte maturation [6]. Similar observations were made by Wang et al. [7], who in addition found delayed primary ossification with irregular distribution of osteoblasts in *Egfr*^−/−^ embryos [7].

Moreover, EGFR knock-in mice where the murine EGFR is replaced by the human counterpart display low EGFR activity in the bone and show impaired endochondral ossification and an increased hypertrophic chondrocyte zone [6]. Similarly, mice with reduced EGFR activity by combined expression of a dominant-negative *Egfr Wa5* allele and deletion of an *Egfr*floxed allele using *Col1a1-Cre* mice (*Col1a1-Cre Egfr*^wa5*/f*^), display bone abnormalities starting around 3 months of age [8]. Mice lacking the membrane-anchored metalloproteinase ADAM17, responsible for cleavage of several membrane-bound cytokines and growth factors including EGFR ligands also develop expanded zones of hypertrophic chondrocytes, and chondrocyte-specific deletion of ADAM17 results in shortened long-bones with increased cartilage mineralization [9].

EGF treatment of WT calvariae increased the proliferation of osteoprogenitor cells and maintained them in an undifferentiated state [10]. Accordingly, *Egfr*^−/−^ osteoblasts show reduced proliferation but elevated differentiation indicating that EGFR is essential during osteoblast maturation [8]. However, the underlying molecular mechanisms has so far not been investigated. It is also unclear whether the bone defects observed in adult mice result from developmental defects or arise later during bone remodeling. The mouse models employed so far have not allowed to investigate this effect, since incomplete EGFR deletion was observed using Col-Cre mice and osteoblast-independent effects of the ubiquitously expressed, dominant-negative Wa5 on other ErbB family members cannot be excluded [8].

Here we investigated the bone phenotype occurring in the first weeks of age in *Egfr*^−/−^ mice and in adult mice in which *Egfr* is conditionally deleted in the osteoblast lineage using *Egfr*^*f*/*f*^ *Runx2*-*Cre* (*Egfr*^ΔOb^) mice. We found that EGFR signaling in osteoblasts negatively regulates IGF-1R/mTOR pathway via ERK1/2 dependent up-regulaion of IGFBP-3 to coordinate differentiation during embryonic and postnatal bone formation.

## RESULTS

### *Egfr*^−/−^ mice show impaired endochondral and intramembranous ossification

**Fig. 1:**
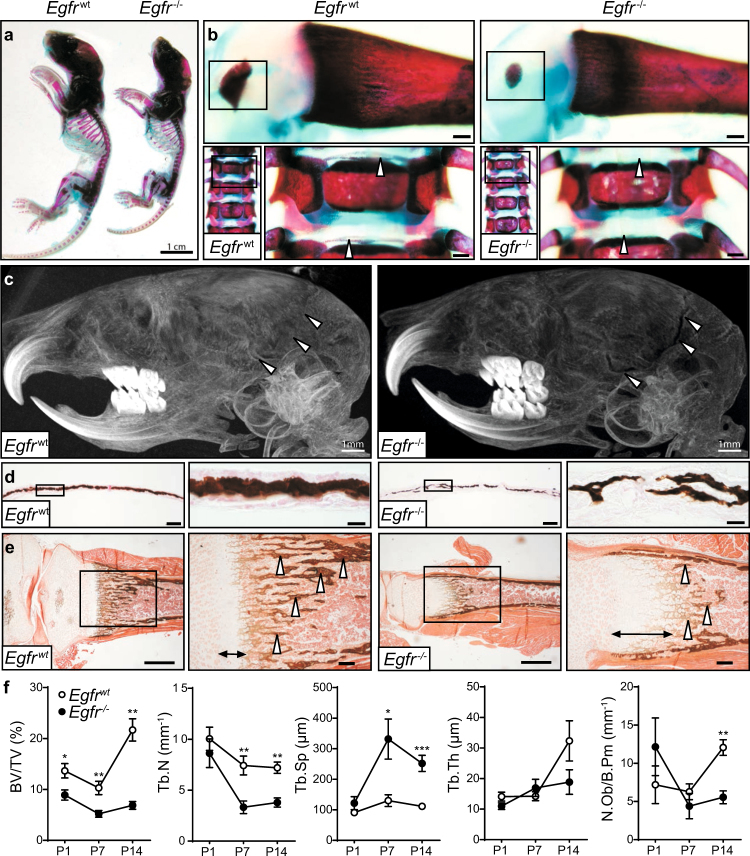
*Egfr*^−/−^ mice are osteopenic. **a** Alcian blue and Alizarin red whole body mount showing skeletal mineralization of *Egfr*^wt^ and *Egfr*^−/−^ mice on postnatal day 7 (P7). **b** Skeletal preparations of WT and KO mice: femur and spine. **c** µCT image of 7-day-old *Egfr*^wt^ (left) and *Egfr*^-/-^ (right) mice **d** Von Kossa staining of *Egfr*^wt^ and *Egfr*^−/−^ calvaria at P7; scales: 100 μm for lower and 20 µm for higher magnification.** e** Von Kossa staining showing calcification of *Egfr*^wt^ and *Egfr*^−/−^ tibiae on P7; scales: 500 μm for lower and and 100 μm for higher magnification. **f** Histomorphometric analysis of *Egfr*^wt^ and *Egfr*^−/−^ tibiae from P1 to P14: Quantification of bone volume/tissue volume (BV/TV), trabecular number (Tb.N), trabecular separation (Tb.Sp), trabecular thickness (Tb.Th) and osteoblast number per bone perimeter (N.Ob/B.Pm). P1: *n* = 6. P7: *n* = 5. P14: *n* = 6 WT, 3 KO mice

We first performed an analysis of the skeleton of *Egfr*^*−*/−^ mice that survived until postnatal day 7 (P7). Bones of *Egfr*^*−*/−^ mice were less mineralized and reduced in length compared to WT littermates (Fig. 1a). Whole-mount body staining revealed reduced centers of secondary ossification in long bones and irregular calcification of vertebral endplates in EGFR-deficient mice (Fig. 1b). Additionally, *Egfr*^−/−^ mice showed reduced mineralization of costal cartilage (Fig. S1a).

While most bones develop by endochondral ossification, the lateral clavicles and parts of the skull are formed by intramembranous ossification, where mesenchymal cells directly differentiate into osteoblasts without chondrocyte involvement [1, 11]. To determine whether the bone phenotype of *Egfr*^−/−^ mice can occur independently of the cartilage defects, we examined skulls of *Egfr*^−/−^ mice. µCT analysis revealed an impaired cranial suture closure on day 14 (Fig. 1c), indicating that EGFR also plays an important role during intramembranous ossification. Furthermore, while straight, well-organized columns of calcified extracellular matrix (ECM) with a clearly delineated border were observed in WTs, these structures were lacking in *Egfr*^−/−^ calvariae (Fig. 1d). Taken together our results show that *Egfr* deletion leads to impaired bone development in newborn mice with defects in both endochondral and intramembranous ossification.

*Egfr*^−/−^ long-bones displayed a low-bone-mass phenotype with less calcified bone and fewer bony trabeculae on P7 (arrowheads; Fig. 1e) and a thickened growth plate (arrows; Fig. 1e). *Egfr*^−/−^ tibiae exhibited thicker zones of ECM located at the cortical sides reaching into the center of the bone (Fig. S1b) indicating that the mineralization process in *Egfr*^−/−^ bones was impaired due to misbalanced deposition of ECM by osteoblasts.

Histomorphometric analyses confirmed that the ratio of bone volume over tissue volume (BV/TV) was significantly lower in *Egfr*^−/−^ mice (Fig. 1f). The trabecular number (Tb.N) was decreased while trabecular separation (Tb.Sp) was increased at P7 and P14, although trabecular thickness (Tb.Th) was not significantly changed (Fig. 1f). While *Egfr*^−/−^ mice were born with osteoblast numbers (N.Ob) comparable to WT levels, their amount was significantly decreased on P14 (Fig. 1f).

### EGFR is essential for osteoblast proliferation and ERK1/2 activation

As osteoblasts are essential for bone mineralization we next focused on the role of EGFR during osteoblastogenesis. We found decreased proliferation of primary pre-osteoblasts lacking the EGFR [6] (Fig. S2a), without any significant differences in the number of apoptotic cells (Fig. S2b). Additionally, *Egfr*^−/−^ osteo-progenitors showed reduced BrdU and Cyclin D1 levels, indicating that EGFR deletion leads to cell autonomous proliferation defects without affecting apoptosis (Figs. S2c, d).

**Fig. 2:**
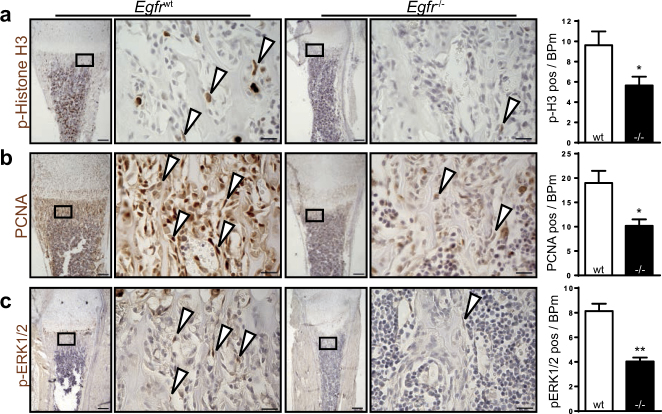
EGFR deletion leads to reduced proliferation and pERK1/2 in bone-lining cells. Representative images and quantifications of IHC stainings on femur sections from 7-day-old *Egfr*^wt^ and *Egfr*^−/−^ mice against (**a**) p-Histone H3 (*n* = 5), (**b**) PCNA (*n* = 5), and (**c**) pERK1/2 (*n* = 3). Scales: 200 µm (lower magnification) and 20 µm (higher magnification)

To confirm that the observed defects are also occurring in vivo, we evaluated the proliferation of bone-lining cells in femoral sections of *Egfr*^−/−^ and *Egfr*^wt^ mice. The number of cells positive for the mitosis marker p-Histone H3 (Fig. 2a) and the S-phase related marker PCNA (Fig. 2b) were significantly reduced in *Egfr*^−/−^ mice indicating that EGFR is crucial for proliferation during bone development.

The ERK pathway, a major EGFR downstream signaling pathway, plays a central role in cell proliferation [12]. Therefore, we analyzed the phosphorylation of ERK1/2 in bone lining cells at P7. *Egfr*^−/−^ mice exhibited significantly reduced numbers of p-ERK1/2 positive cells on their trabecular bone (Fig. 2c), suggesting that the proliferation defects during bone development are based on impaired ERK1/2 activation.

### Osteoblast-specific deletion of EGFR leads to bone defects

**Fig. 3:**
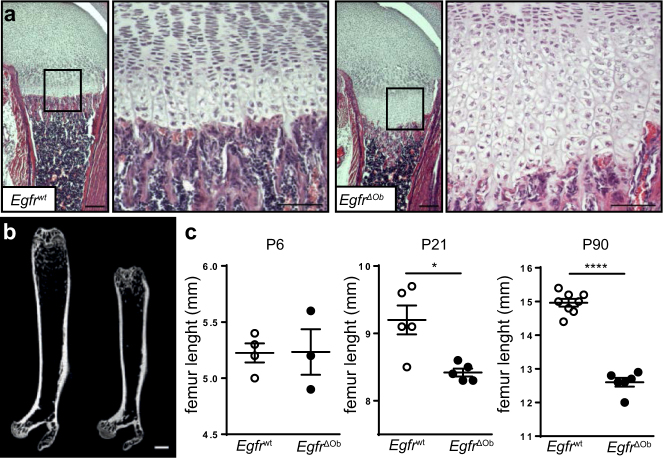
*Egfr*^ΔOb^ mice phenocopy the bone phenotype of *Egfr*^−/−^ mice. **a** H&E stainings showing distal femurs with increased zone of hypertrophic chondrocytes in 6-day-old *Egfr*^ΔOb^ mice; scales: 200 µm (lower magnification) and 100 µm (higher magnification). **b** µCT image of femurs from 3-months old *Egfr*^wt^ and *Egfr*^ΔOb^ littermate; scale: 1 mm. **c** Quantification of femur length of *Egfr*^wt^ and *Egfr*^ΔOb^ mice with indicated age

To address whether the bone phenotype in *Egfr*^−/−^ mice is due to cell-autonomous defects in osteoblasts, *Egfr*^f/f^ mice were crossed to an osteoblast-specific Cre line (*Runx2-Cre*), to generate *Egfr*^*f*/*f*^ *Runx2-Cre* (*Egfr*^ΔOb^) mice [13]. Complete deletion of EGFR was confirmed by Western Blot in cultured osteoblasts and by IHC in long bones (Figs. S3a, b). As shown by qRT-PCR from RNA isolated from bone and cartilage of *Egfr*^wt^ and *Egfr*^ΔOb^ femurs, deletion of *Egfr* was restricted to bone tissue, but not cartilage (Fig. S3c). *Egfr*^ΔOb^ mice developed normally without any significant differences in overall body length (Fig. S3d). On P6 the zone of hypertrophic chondrocytes of *Egfr*^ΔOb^ mice was significantly increased, comparable to *Egfr*^−/−^ mice (Fig. 3a). Importantly, *Egfr*^ΔOb^ mice showed reduced length of long bones which was significant by P21 and became more severe with age (Figs. 3b, c). These results demonstrate that EGFR signaling in osteoblasts is essential for proper bone development.

### Adult *Egfr*^ΔOb^ mice develop a low-bone-mass phenotype

**Fig. 4:**
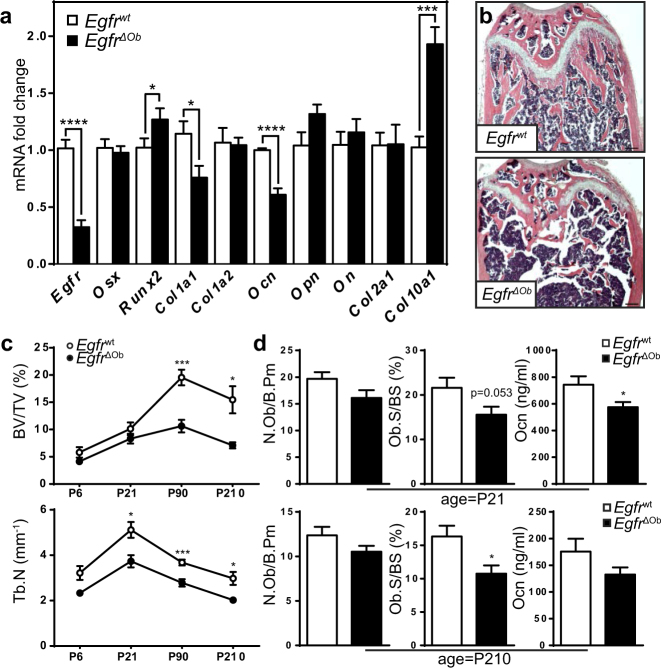
*Egfr*^ΔOb^ mice show severe bone defects. **a** qRT-PCR analysis: RNA isolated from whole femurs of 21-day-old *Egfr*^wt^ and *Egfr*^ΔOb^ mice; *n* = 7 WT, 9 ΔOb. **b** H&E stainings showing distal femurs of 3-months-old *Egfr*^wt^ and *Egfr*^ΔOb^ mice; scales: 200 µm. **c** Histomorphometric analysis of WT/ΔOb long-bones at P6, P21 P90 and P210: Quantification of bone volume/tissue volume (BV/TV) and trabecular number (Tb.N). P6: *n* = 4 WT, 3 ΔOb. P21: *n* = 5. P90: *n* = 8 WT, 6 ΔOb. P210: *n* = 4 WT, 6 ΔOb mice. **d** Osteoblast number (N.Ob/B.Pm) and surface on the trabecular bone (Ob.S/BS) at P21 (*n* = 7 WT, 10 ΔOb) and P210 (*n* = 6 WT, 8 ΔOb) and Osteocalcin as measured by ELISA in Serum at P21 (*n* = 4 WT, 6 ΔOb) and P210 (*n* = 5 WT, 9 ΔOb)

The augmented zone of hypertrophic chondrocytes was accompanied by increased expression of the hypertrophic chondrocyte marker *Col10a1* in long bones of *Egfr*^ΔOb^ mice on P21 (Fig. 4a). Significantly elevated Runt-related transcription factor-2 (*Runx2)* mRNA levels together with reduced Colagen1a1 (*Col1a1*) mRNA and reduced Osteocalcin (*Ocn*) mRNA and protein levels (Figs. 4a, S4a) indicate that EGFR deletion in osteoblasts leads to impaired mineralization due to premature differentiation of osteoprogenitors. Histomorphological analysis revealed a progressive, low-bone-mass phenotype with decreased bone volume and trabecular number in adult *Egfr*^ΔOb^ mice (Figs. 4b, c). Additional trabecular bone markers further showed reduced trabecular thickness and increased spacing in *Egfr*^ΔOb^ mice (Fig. S4b). Less osteoblasts on the trabecular bone and reduced osteocalcin serum levels (Fig. 4d) indicate that the low-bone-mass is based on osteoblast defects.

To exclude that EGFR in osteoblasts indirectly affects osteoclastogenesis, osteoclast-specific markers were analyzed in long bones and serum. No significant differences in osteoclast number could be detected neither in young nor in adult *Egfr*^ΔOb^ mice. Furthermore, *Egfr*^ΔOb^ mice did not show any differences of the serum biomarker for bone resorption C-terminal telopeptide (CTX-1) (Fig. S4c).

Additionally, we assessed whether EGFR directly affects osteoclast development by breeding *Egfr*^*f*/*f*^ mice to *LysM-Cre* mice that express Cre recombinase in the myeloid lineage (*Egfr*^ΔOc^). Osteoclasts isolated from *Egfr*^ΔOc^ mice showed reduced EGFR protein levels (Fig. S4d), but did not display any bone defects nor differences in the number of osteoclasts in trabecular bones or serum CTX-1 (Fig. S4e). Bone-marrow derived pre-osteoclasts from *Egfr*^−/−^ mice did not show any significant differences in their ability to form osteoclasts in vitro (Fig. S4f). Finally, OC number in trabecular bones and serum CTX-1 levels were not altered in *Egfr*^-/-^ mice (Fig. S4g) indicating that lack of EGFR does not affect osteoclastogenesis.

### Enhanced differentiation of *Egfr*^−/−^ osteoblasts correlates with IGF-1R/mTOR activation

**Fig. 5:**
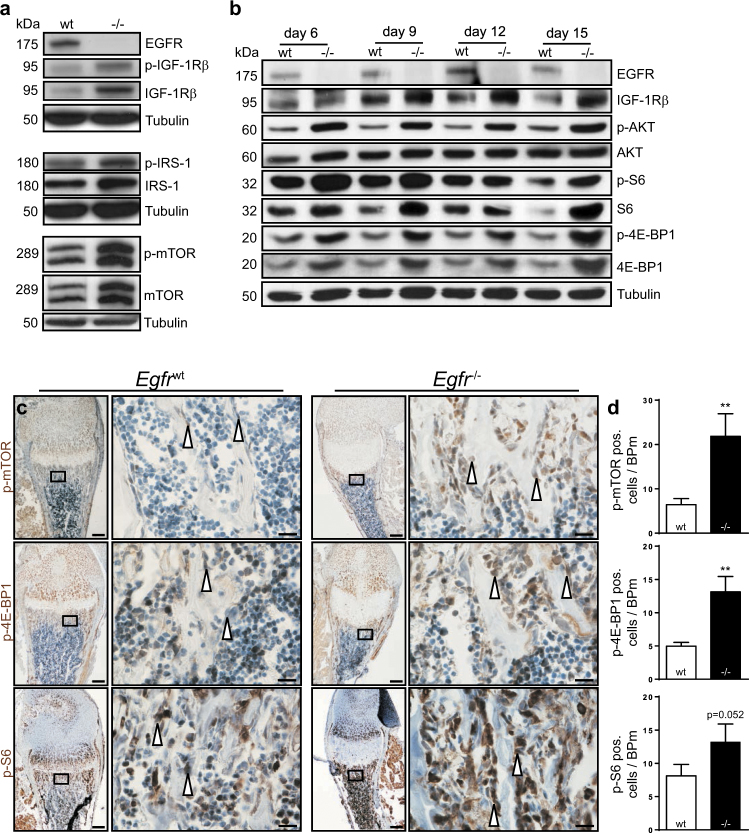
Enhanced differentiation correlates with mTOR-signaling. **a** Western blot analysis of *Egfr*^wt^ and *Egfr*^−/−^ osteoblasts under differentiation conditions; isolated on differentiation day 14. **b** Western blot analyses of *Egfr*^wt^ and *Egfr*^−/−^ osteoblasts under differentiation conditions on days 6, 9, 12, and 15. **c** Immunohistochemical staining of p-mTOR, p-4E-BP1 and p-S6 on trabecular bone sections from distal femurs of P7 *Egfr*^wt^ and *Egfr*^−/−^ littermates; scales: 200 µm (lower magnification) and 20 µm (higher magnification). **d** Quantification of IHC staining, shown as positive cells per bone perimeter (B.Pm); *n* ≥ 3

Once confirmed that the defects are primarily in the osteoblast lineage, we employed primary osteoblasts from *Egfr*^wt^ and *Egfr*^−/−^ mice to investigate the underlying molecular mechanism. As osteoblasts from *Egfr*^−/−^ mice display enhanced differentiation [6] and the IGF-1R pathway was shown to play a central role during osteoblast differentiation [14], we investigated whether EGFR regulates bone development by interacting with the IGF-1R signaling pathway. We detected elevated levels of total and phosphorylated IGF1Rβ in differentiated osteoblasts isolated from *Egfr*^−/−^ mice (Fig. 5a). Furthermore, *Egfr*^−/−^ osteoblasts showed increased total and phosphorylated protein levels of the IGF-1R adapter protein insulin receptor substrate 1 (IRS-1) and its downstream target mTOR (Fig. 5a). Importantly, IGF-1R/IRS1/mTOR up-regulation was ligand independent as the levels of IGF-1 and IGF-2 were not altered (Fig. S5a).

To investigate the kinetics of mTOR activation we next analyzed multiple time points during osteoblast differentiation. IGF-1R/mTOR-pathway proteins were consistently present at higher levels and were hyper-phosphorylated during differentiation in *Egfr*^−/−^ osteoblasts indicating that IGF-1R/mTOR-signaling remained elevated throughout the whole culture period (Fig. 5b).

IHC staining on femur sections of WT and EGFR-deficient mice at P7 revealed that the mTOR-signaling pathway was also altered in vivo. In line with the in vitro findings, significantly increased phosphorylation of mTOR and its main downstream targets 4E-BP1 and S6 protein were observed in *Egfr*^−/−^ long-bones (Figs. 5c, d). Additionally, *Egfr*^ΔOb^ mice also showed reduced p-S6 protein levels in bone-lining cells indicating that this activation depends on osteoblastic EGFR signaling (Figs. S5b, c).

### Interplay between EGFR- and IGF-1R-pathways in osteoblast differentiation

**Fig. 6:**
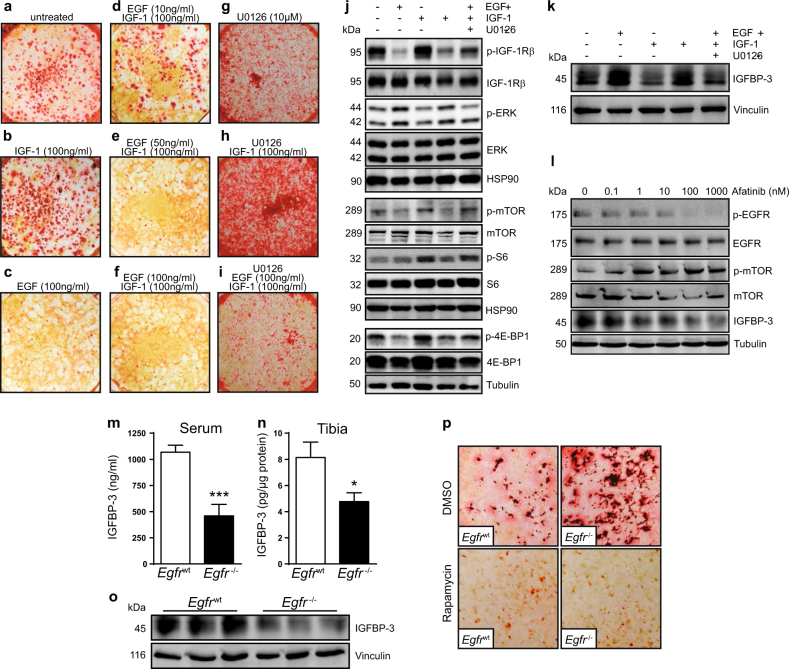
Differentiation in WT osteoblasts is mediated by specific components of the IGF-1R-pathway and inhibited by EGF. **a**–**i** Alizarin red staining of WT osteoblasts after 21 days (D21) in culture under differentiation conditions (+AA, βGP) with EGF, IGF-1, and/or U0126 over the whole culture period. Pictures taken from a 6-well plate. **j**, **k** Western blot analysis of differentiated WT osteoblasts (D21) cultured with EGF (100 ng), IGF-1 (100 ng) and/or U0126 (10 µM). **l** Western Blot analysis of undifferentiated osteoblast precursor cells cultured in αMEM + 10%FCS after 4 h treatment with indicated concentrations of Afatinib. **m** IGFBP-3 protein levels in serum (*n* = 7) and **n** whole tibia lysates (*n* = 4) of 7-day old *Egfr*^wt^ and *Egfr*^−/−^ littermates. **o** Western Blot analysis of whole tibia protein lysates isolated from 7-day old *Egfr*^wt^ and *Egfr*^−/−^ littermates. **p** Alizarin red staining of differentiated WT and *Egfr*^−/−^ osteoblasts (D21) cultured with vehicle (DMSO) or Rapamycin (10 nM). Stained with alizarin red

To analyze the cross-talk between EGFR and IGF-1R-signaling during osteoblast differentiation, WT osteoblasts were cultured under differentiation-inducing conditions together with IGF-1, EGF and/or the ERK1/2 inhibitor U0126. At day 21 bone nodule formation was assessed as a functional read-out for differentiation. Mineralization was enhanced by IGF-1 treatment (Figs. 6a, b) and completely abolished by EGF (Fig. 6c). Addition of EGF was able to suppress IGF-1 induced differentiation in a dose-dependent manner with complete inhibition at 100 ng/ml (Figs. 6d–f). IGF-1 induced differentiation was further increased when ERK1/2 signaling was blocked by U0126 (Figs. 6g, h). ERK inhibition together with EGF and IGF-1 stimulation rescued the EGF-induced hypo-differentiation phenotype resulting in normalized bone nodule formation comparable to untreated controls (Fig. 6i). Taken together our results show that IGF-1R signaling enhances, whereas EGFR signaling inhibits osteoblast differentiation and that EGFR signaling dominates by negatively regulating IGF-1R via ERK1/2.

To dissect the underlying molecular mechanism we analyzed the activation of EGFR and IGF-1R downstream proteins in differentiated WT osteoblasts cultured for 21 days in the presence of EGF / IGF-1 and U0126. EGF treatment prevented phosphorylation of IGF-1Rβ with reduced activation of the mTOR/S6/4E-BP1 pathway, whereas IGF-1 induced the phosphorylation of IGF-1Rβ/mTOR/S6/4E-BP1 (Fig. 6j). When osteoblasts were cultured with both growth factors, activation was again reduced suggesting that EGFR signaling is able to block differentiation via IGF-1Rβ inhibition. Importantly, EGF-induced downregulation of the IGF-1Rβ pathway was partly restored when ERK1/2 was blocked, indicating that EGFR negatively regulates differentiation by down-regulating IGF-1Rβ/mTOR signaling via ERK1/2 (Fig. 6j). No differences in insulin receptor β (IRβ) phosphorylation could be detected indicating that EGF stimulation exclusively downregulates IGF-1R without affecting IRβ activation (Fig. S6a).

To prove that reduced activation of IGF-1Rβ is a direct consequence of EGF stimulation, we cultured WT osteoblasts under differentiation conditions for 21 days, starved them for 24 h and stimulated for 10 min with EGF or IGF-1. As expected, EGF treatment induced a strong activation of ERK1/2 and at the same time reduced the phosphorylation of IGF-1Rβ whereas IGF-1 stimulation did not affect ERK1/2 signaling (Fig. S6b).

To investigate the mechanism how EGFR signaling suppresses IGF-1R/mTOR signaling we next analyzed IGFBP-3 levels, as IGFBP-3 is known to modulate and repress IGF-1R signaling [15, 16]. Moreover, it has been shown that EGFR directly regulates IGFBP-3 in primary esophageal cells [17]. We found elevated IGFBP-3 levels in osteoblasts cultured together with EGF or with EGF and IGF-1 whereas IGF-1 alone had no effect (Fig. 6k). Importantly, IGFBP-3 up-regulation was a direct consequence of ERK1/2 signaling, as additional treatment with the ERK1/2 inhibitor U0126 normalized EGF-induced IGFBP-3 levels (Fig. 6k). In contrast, EGFR inhibition with Afatinib led to a dose-dependent decrease in IGFBP-3 protein levels along with increased p-mTOR phosphorylation in osteoblast precursors (Fig. 6l). In addition, IGFBP-3 was also strongly reduced in the supernatant of osteoblast precursor cells after 48 h treatment with EGFR inhibitor as compared to DMSO treated controls (Fig. S6c).

In line with our in vitro results, we also found significantly reduced IGFBP-3 in the serum of *Egfr*^−/−^ and *Egfr*^ΔOb^ mice (Figs. 6m, S6d) indicating that EGFR signaling in osteoblasts is essential for IGFBP-3 production. IGFBP-3 levels were also reduced in whole tibia protein lysates of *Egfr*^−/−^ mice, as revealed by both ELISA and western blot analysis (Figs. 6n, o). These results demonstrate that EGFR is required for IGFBP-3 production and suppression of IGF-1R/mTOR activation thus providing a mechanistic link between EGFR and IGF-1R signaling and osteoblast differentiation.

To further show that the hyper-differentiation phenotype of *Egfr*^−/−^ osteoblasts is indeed a consequence of elevated mTOR activation we next inhibited mTOR in differentiating osteoblasts using rapamycin. Bone nodule formation was strongly reduced in the presence of rapamycin (Fig. 6p). Upon rapamycin treatment, phosphorylation of the mTOR downstream proteins 4E-BP1 and S6 was down-regulated in *Egfr*^−/−^ cultures similarly to WT osteoblasts (Fig. S6e) demonstrating that the increased differentiation in *Egfr*^−/−^ osteoblasts can be prevented by mTOR-inhibition.

Taken together our data provide evidence that EGFR controls osteoblasts differentiation via ERK-dependent IGFBP-3 up-regulation, which ensures proper osteoblast maturation by controlling IGF-1R/mTOR signaling.

### mTOR inhibition partially rescues bone phenotype of *Egfr*^−/−^ embryos

**Fig. 7:**
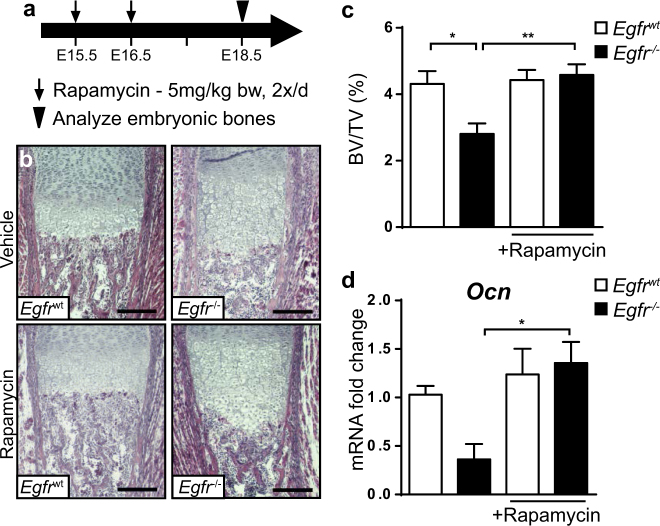
mTOR inhibition partially rescues bone phenotype of *Egfr*^−/−^ mice. **a** Timeline showing experiment outline for in utero Rapamycin treatment. Pregnant mice were subcutaneously injected with 5 mg Rapamycin per kg bodyweight or Injection-vehicle twice a day on E15.5 and E16.5 **b** H&E staining of trabecular bone sections showing distal femurs of *Egfr*^wt^ and *Egfr*^−/−^ embryos on E18.5 after Rapamycin/vehicle treatment; scale: 200 µm. **c** Quantification of bone volume relative to total volume (BV/TV) after rapamycin/vehicle treatment; *n* = 7 WT, 6 KO mice for vehicle and 6 WT, 8 KO mice for rapamycin treatment; 5 mothers per treatment group. **d** mRNA expression levels of *Osteocalcin* (*Ocn*) in femurs of E18.5 mice measured by qRT-PCR; *n* = 9 WT, 5 KO mice for vehicle and 7 WT, 5 KO mice for rapamycin treatment

We next analyzed whether mTOR inhibition during embryonic development, when mineralization starts, can normalize the bone defects in EGFR-deficient mice. Pharmacological inhibition of mTOR during gestation has previously been reported not to cause any bone-specific side effects in mice [18]. We injected pregnant females from EGFR heterozygous intercrosses with rapamycin or vehicle twice a day on E15.5 and on E16.5 and analyzed embryonic bones at E18.5 (Fig. 7a). Rapamycin treatment was not teratogenic nor did it affect litter size or viability of pups (Fig. S7a). Inhibition of mTOR signaling pathway was confirmed by p-S6 IHC staining on femurs of fetuses obtained from rapamycin or vehicle-treated mothers (Fig. S7b).

We could not observe any effect on hypertrophic chondrocyte zone in embryonic *Egfr*^−/−^ bones after rapamycin treatment (Fig. S7c), which is in line with our hypothesis that the hypertrophic chondrocyte phenotype is not responsible for the impaired bone development. However, chemical inhibition of mTOR increased the zone of hypertrophic chondrocytes in WT animals (Fig. S7c) without affecting *Egfr* expression levels in long-bones (Fig. S7d).

Importantly, after rapamycin treatment bones of *Egfr*^−/−^ embryos showed BV/TV comparable to WT mice (Figs. 7b, c). Furthermore, *Osteocalcin* mRNA levels in femurs of *Egfr*^-/-^ embryos from rapamycin-injected mothers were also normalized (Fig. 7d). In addition, rapamycin treatment also normalized the ratio between *Runx2* and *Osteocalcin* mRNA expression in bones of *Egfr*^−/−^ embryos (Figs. S7e, f) providing evidence that EGFR signaling suppresses mTOR during bone formation to prevent early maturation of osteoprogenitor cells to ensure the development of functional osteoblasts.

## Discussion

In the present study, we show that EGFR-deficient mice suffer from a complex bone phenotype with decreased bone mass, which starts before birth and persists to adulthood. Moreover, deleting EGFR specifically in the osteoblast or osteoclast lineage demonstrates that EGFR in the osteoblast lineage is essential for adequate bone development.

Histological analyses revealed an enlarged zone of hypertrophic chondrocytes, which could be the reason for the subsequent bone defects. However, we show that both endochondral as well as intramembranous ossification is defective in the absence of EGFR. Since intramembranous ossification does not involve chondrocyte differentiation and cartilage formation, our results suggest that the osteoblast and bone defects are unlikely to result from chondrocyte defects. Therefore, EGFR signaling seems to be required cell-autonomously in osteoblasts. Long-bones of mice with osteoblast-specific deletion of EGFR showed elevated *Runx2* with reduced *Colagen1a1* and *Osteocalcin* expression levels revealing an important role of EGFR during mineralization. This finding also reflects results from published in vitro experiments suggesting that a major function of the EGFR is to maintain a pool of osteoprogenitor cells by downregulating Runx2 and Osterix in order to prevent premature differentiation [19]. Despite the fact that *Egfr*^−/−^ osteoblasts showed elevated mineralization in vitro, both *Egfr*^−/−^ and *Egfr*^ΔOb^ mice are osteopenic. This apparent discrepancy might be due to the fact that osteoprogenitor cells lacking the EGFR, which display proliferation defects, cannot form sufficient numbers of osteoblasts to guarantee proper maturation and ossification in vivo.

We identified the mTOR-pathway as a positive regulator of osteoblast differentiation that is suppressed by EGFR signaling. In the absence of EGFR, IGF-1R/mTOR signaling is up-regulated due to reduced IGFBP-3 signaling leading to accelerated osteoblast differentiation thus not allowing a sufficient number of osteoprogenitor cells to accumulate to form proper bones. Under normal physiological conditions EGFR/ERK-mediated IGFBP-3 is essential to suppress IGF-1R/mTOR in order to ensure efficient osteoblasts maturation.

Many possible interactions between IGF-1R and EGFR have been identified [20]. Cancer cells acquire resistance against EGFR inhibitor treatment via loss of IGFBP-3, which activates the IGF-1R signaling pathway [21, 22]. A tight regulation of IGFBP-3 signaling is not only essential for cancer treatment but also during bone development as shown by both *Igfbp3* transgenic and knock-out mouse models. Long-bones of *Igfbp3* transgenic mice overexpressing human *IGFBP-3* demonstrate reduced trabecular and cortical bone density [23]. *Igfbp3*^−/−^ mice, on the other hand, develop a low-bone-mass phenotype comparable to *Egfr*-deficient mice comprising reduced trabecular bone volume and number with increased trabecular separation [24]. In agreement with our data, a link between EGFR and IGFBP-3 has also been described for primary human esophageal cells and esophageal squamous cell carcinomas indicating that EGFR indeed directly regulates IGFBP-3 [17].

The mTOR-pathway plays an important role during development by regulating cell survival, growth, differentiation and autophagy [25]. Recently, rapamycin-induced autophagy was shown to increase the number of osteoblasts and the mineralized area in fracture calluses of rats during bone fracture healing [26]. mTOR signaling has also been linked to other bone-related diseases like osteoarthritis (OA). Patients suffering from OA show increased mTOR protein and mRNA levels in affected joints [27]. Additionally, rapamycin treatment or deletion of mTOR in chondrocytes reduced the severity of experimental OA in mice [27, 28]. Reduced EGFR signaling, on the other hand, leads to a worse progression of experimental OA due to increased cartilage destruction in gefitinib-treated mice [29] and subchondral bone plate thickening with increased joint pain in genetically modified (*Egfr*^wa5/f^ *Col2-Cre*) animals [30]. These findings suggest that EGFR might not only negatively regulate the mTOR-pathway during bone development, but also during OA progression. Further studies are needed to investigate the impact of EGFR signaling on mTOR activation in bone-related diseases.

Mice with osteoblast-specific IGF-1R deletion display mineralization defects [31, 32]. mTOR signaling pathway activation via IGF-1 has been reported to play a major role in bone development by regulating osteoblast differentiation in adult mice [32]. Moreover, osteoblast-specific deletion of TSC2, a negative regulator of the mTOR pathway, leads to elevated mTOR signaling with increased bone formation starting around 6 weeks after birth. Interestingly, three weeks after birth these mice showed an osteopenic-like phenotype with significantly increased trabecular separation, reduced bone volume to tissue volume and reduced number of trabecles [33]. As *Egfr*^−/−^ mice also exhibit an osteopenic bone phenotype with elevated mTOR expression in osteoblasts, we hypothesize that up-regulation of mTOR pathway might inhibit bone formation during embryonic and early postnatal development, whereas it induces bone mineralization in older animals. Consistently, treatment of pregnant dams with rapamycin largely rescued the low bone mass phenotype of *Egfr*^−/−^ embryos.

In summary, we demonstrate that impaired proliferation and enhanced differentiation of osteoblasts is responsible for the osteopenia and irregular mineralization in bones of *Egfr*^−/−^ and *Egfr*^ΔOb^ mice. The bone defects of *Egfr*^−/−^ mice are not restricted to endochondral ossification, since mineralization defects are also apparent in skulls of *Egfr*^−/−^ pups. Therefore, defective osteoblast maturation very likely is the driving force for the mineralization defects in *Egfr*^−/−^ mice. We identified the mTOR-pathway as a positive regulator of osteoblast differentiation, suppressed by EGFR/ERK/IGFBP-3-signaling and hyper-activated in its absence via IGF-1R. Future studies will address whether the cross-talk between these important signaling pathways is also operating in other tissues and under pathological conditions.

## Materials and methods

### Mice

*Egfr*^−/−^ mice have been described previously [4]. *Egfr*^*∆*Oc^ mice were generated by breeding *Egfr*^*f*/*f*^ mice [34] to *LysM-Cre* [35] transgenic mice. *Egfr*^*∆*Ob^ mice were generated by crossing *Egfr*^*f*/*f*^ mice with *Runx2-Cre* [13] transgenic mice (kindly provided by Jan Tuckermann, University Ulm). Only male *Egfr*^*∆*Ob^ and littermate controls (*Egfr*^*f*/*f*^, *Egfr*^*f*/+^ or *Egfr*^*f*/+^ *Runx2-Cre*) with a C57BL/6 genetic background were used for experiments. Genotyping was performed as previously described [4, 35, 13]. Mice were kept in the animal facility of the Medical University of Vienna in accordance with institutional policies and federal guidelines. All animal experiments conducted were compliant with federal laws and guidelines of the Medical University of Vienna.

### Whole mount stainings, histomorphometry, immunohistochemistry

Mice were sacrificed at indicated time points. Whole mount stainings were performed as described previously [36]. For histological stainings, bones were fixed in 4% PBS-buffered formaline and embedded either in paraffin or methylmetacrylate. 5 μm paraffin sections were used for H.E.-stainings after decalcification in 0.5 M EDTA or uncalcified for Von-Kossa stainings (calvaria); methylmetacrylate was used for Von-Kossa stainings (long bone) and for Movat-stainings (osteoid). Histomorphometry was performed with Movat and/or H&E-stainings according to the standardized protocols of the American Society for Bone and Mineral Research [37] on the Osteo-measure system (Osteometrix) in a blinded fashion. Immunohistochemistry was performed on 4 µm formalin-fixed paraffin embedded and decalcified femur sections. Primary antibodies (for a full list see Table S1) were incubated overnight at 4 °C followed by HRP-based immunoreactivity detection (CST). Non-specific binding was blocked by applying TBS-T containing 2% BSA and 5% normal goat serum. Quantifications of IHC stainings were performed in a blinded fashion by counting positive cells on the trabecular bone surface and results are shown as positive cells per bone perimeter.

### Primary osteoblast cultures

Osteoblasts were cultured in α-MEM containing ribonucleosides and deoxyribonucleosides (GlutaMAX, Sigma) and 10% FBS (Autogen Bioclear). Primary osteoblasts were isolated from calvariae of neonatal mice (P1-P7) as previously described [38] and seeded at a density of 5.000 cells/cm^2^. For differentiation, ascorbic acid (50 μg/ml) and β-glycerolphosphate (10 mM) were added to the culture medium. Bone nodules were stained at differentiation day 21 using Alizarin Red (Sigma). For BrdU stainings, osteoblasts were cultured until 70% confluency and incubated with 10 µM BrdU (Roche) for 4 h, before fixation with 70% ethanol and staining with an anti-BrdU antibody according to the manufacturer’s instructions (Becton Dickinson). Rapamycin (Wyeth), EGF (Roche) and IGF-1 (Promega) were used in concentrations indicated in the respective figure legends.

### Primary osteoclast cultures

For osteoclast isolation, bone marrow cells were harvested from long-bones of 8 week old mice. Cells were cultured overnight in α-MEM containing 10% FBS. Non-adherent cells were harvested, counted and seeded in 6-well plates (1.0 × 10^6^ cells/well) with M-CSF (50 ng/ml). 48 h later RANKL (50 ng/ml) was added to induce differentiation for additional 96 h.

### Total RNA isolation, Real-time qRT-PCR analysis

Total RNA from osteoblasts and whole bone was isolated using peqGOLD TriFast reagent (Peqlab) or RNeasy Kit (Qiagen). cDNA synthesis was performed with ProtoScript II Reverse Transcriptase (NEB) according to the manufacturer’s instructions. Real-time qRT-PCR was performed using the Power SYBR Green Master Mix (Thermo Fisher Scientific) together with the Applied Biosystems 7500 Fast Real-Time PCR System (Thermo Fisher Scientific) using the following primers: Collagen type 1 alpha 1 *(Col1a1)* 5′-ACCTGGTCCACAAGGTTTCC-3′ and 5′-GACCCATTGGACCTGAACCG-3′; Collagen type 1 alpha 2 (*Col1a2*) 5′-GGTCCAAGAGGAGAACGTGG-3′ and 5′-TGGGACCTCGGCTTCCAATA-3′; Collagen type 2 alpha 1 (*Col2a1*) 5′-GGCCAGGATGCCCGAAAATTA-3′ and 5′-CGCACCCTTTTCTCCCTTGT-3′; Collagen type 10 alpha 1 (*Col10a1*) 5′-CATCTCCCAGCACCAGAATC-3′ and 5′-GCTAGCAAGTGGGCCCTTTA-3′; Epidermal growth factor receptor (*Egfr*) 5′-TTGGAATCAATTTTACACCGAAT-3′ and 5′-GTTCCCACACAGTGACACCA-3′; Osteocalcin *(Ocn)* 5′-AGACTCCGGCGCTACCTT-3′ and 5′-CTCGTCACAAGCAGGGTTAAG-3′; Osteonectin (On) 5′-TCTCAAAGTCTCGGGCCAAC-3′ and 5′-ATGCAAATACATCGCCCCCT-3′; Osteopontin (*Opn*) 5′-CTGGCTGAATTCTGAGGGACT-3′ and 5′-TTCTGTGGCGCAAGGAGATT-3′; Osterix (*Osx*) 5′-TGCCTGACTCCTTGGGACC-3′ and 5′-TAGTGAGCTTCTTCCTCAAGCA-3′; Runt-related transcription factor 2 *(Runx2)* 5′-GCCGGGAATGATGAGAACTA-3′ and 5′-GGACCGTCCACTGTCACTTT-3′; Expression levels were standardized to the primer set specific for TATA-binding protein *(Tbp)*: 5′-GGGGAGCTGTGATGTGAAGT-3′ and 5′-CCAGGAAATAATTCTGGCTCAT-3′.

### Western blot analysis

Western blot analysis was performed as previously described [39]. For a full list of the antibodies used, please see Table S1.

### Enzyme-linked immunosorbent assay (ELISA)

Mouse IGF-1 (Quantikine, R&D Systems) and IGF-2 (RayBiotech) Immunoassays were performed according to manufacturer’s instructions with 48 h-old supernatants collected from osteoblast cultures on differentiation day 14. Osteocalcin (Alfa Aesar) and CTX-1 Elisa (RatLaps, IDS Immunodiagnostic Systems) were performed according to the manufacturer’s instructions with serum isolated from male mice at p21 and p210. Mouse IGFBP-3 Elisa (R&D Systems) was performed with 48 h-old supernatants collected from osteoblast cultures on differentiation day 21 or from undifferentiated osteoblast precursors. Serum IGFBP-3 levels where analyzed in serum isolated from p7 and p210 mice. For IGFBP-3 quantification in whole tibia protein lysates from p7 mice, 20 µg protein/well were applied after Bradford-based protein measurement (Bio-Rad).

### Rapamycin treatment

Rapamycin (Sigma) was diluted in injection vehicle containing 10% PEG-400 and 17% Tween-80 in 1 × PBS. Mice were randomly assigned into two groups and injected every 12 h between E15.5 and E16.5 either with 5 mg Rapamycin per kg bw in 200 µl injection vehicle or with 200 µl injection vehicle alone according to a published protocol [18].The investigators were not blinded during the experiment.

### Statistical methods

Sample size calculation: For in vivo treatment experiments a minimum of six embryos per group were considered, which ensures a 90% power to detect a difference in means of 2 standard deviations at the significance level of 0.05. Based on the central limit theorem, we can assume a normal distribution of mean values even if the underlying variable is not perfectly normally distributed. Unless otherwise stated experiments were performed at least 2 times and data are shown as mean ± s.e.m. For analyses of IHC and qRT-PCR data, univariable comparisons of expression values between groups were analyzed by unpaired two-tailed Student’s *t*-test with *f*-test to ensure comparable variances between the groups. For analysis of hypertrophic chondrocyte zone, BV/TV and qRT-PCR analysis after Rapamycin treatment, one-way ANOVA was applied. A *p*-value below 0.05 was considered statistically significant and was marked with a star (*), *p* < 0.01 with 2 stars (**), *p* < 0.001 with 3 stars (***) and *p* < 0.0001 with 4 stars (****). For analyses, SAS for Windows 9.1.3 (The SAS Institute, Inc., Cary, North Carolina, USA) and Prism 6 (GraphPad) were used.

## Electronic supplementary material

Supplemental Figures S1-7

Supplemental Table S1

Supplemental Figure and Table Legends
