# Supplementary figures and images for: EGFR controls bone development by negatively regulating mTOR-signaling during osteoblast differentiation
Source: Cell Death Differ. 2018 Feb 14;25(6):1094–106. doi: 10.1038/s41418-017-0054-7 (PMC5988706; doi:10.1038/s41418-017-0054-7)

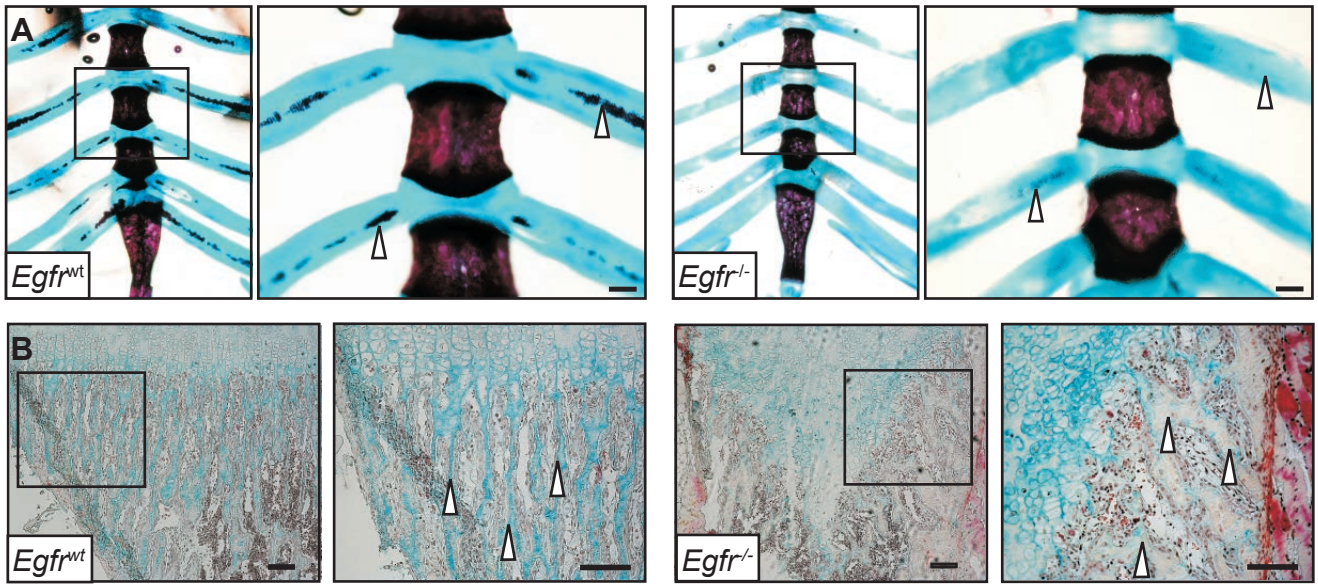

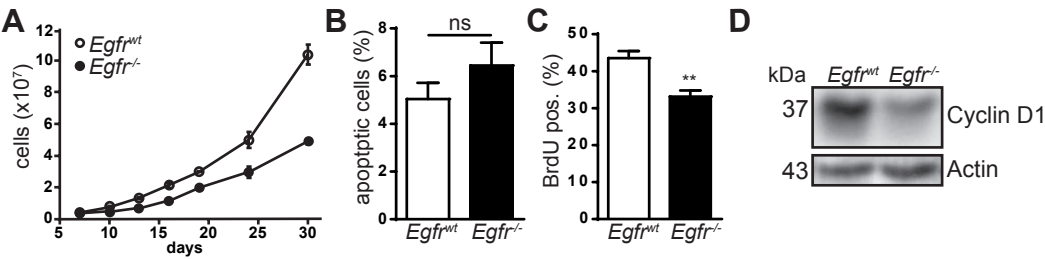

**A**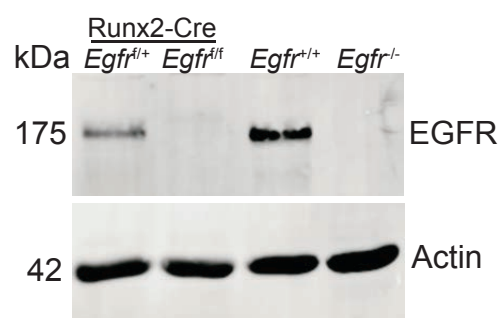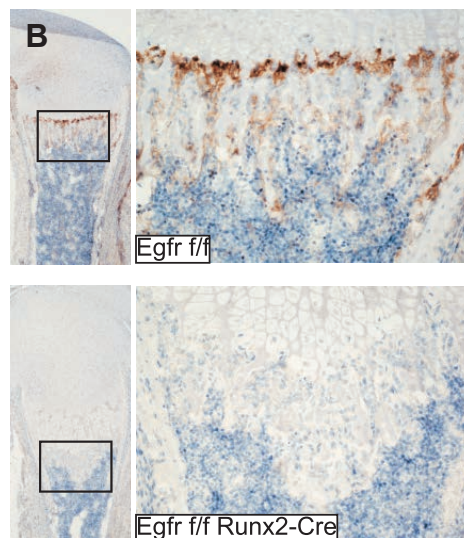**C**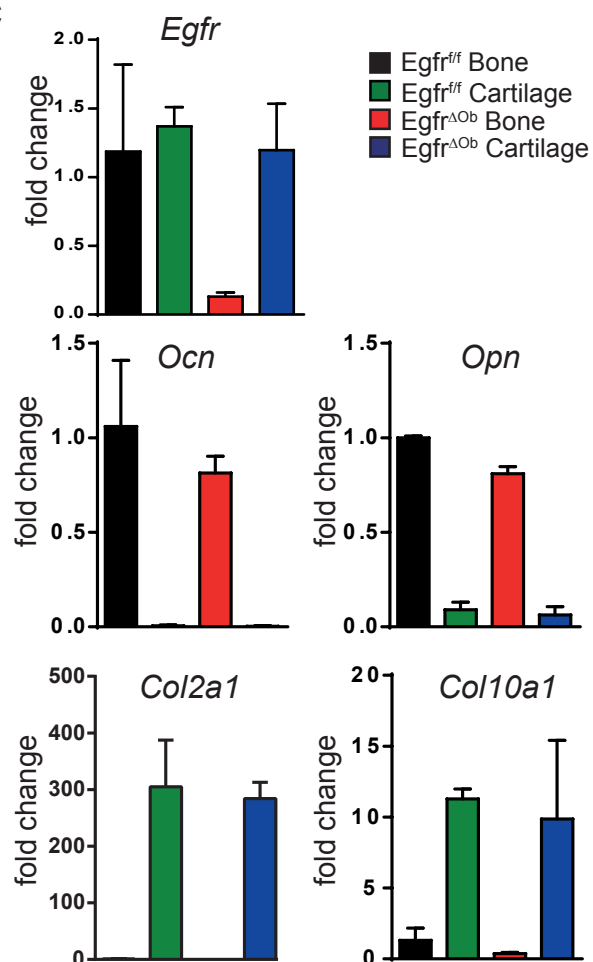**D**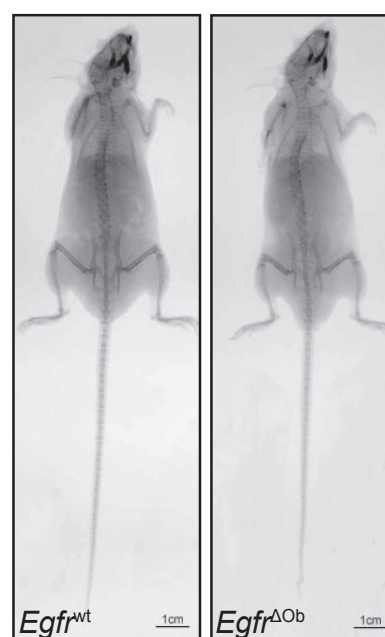

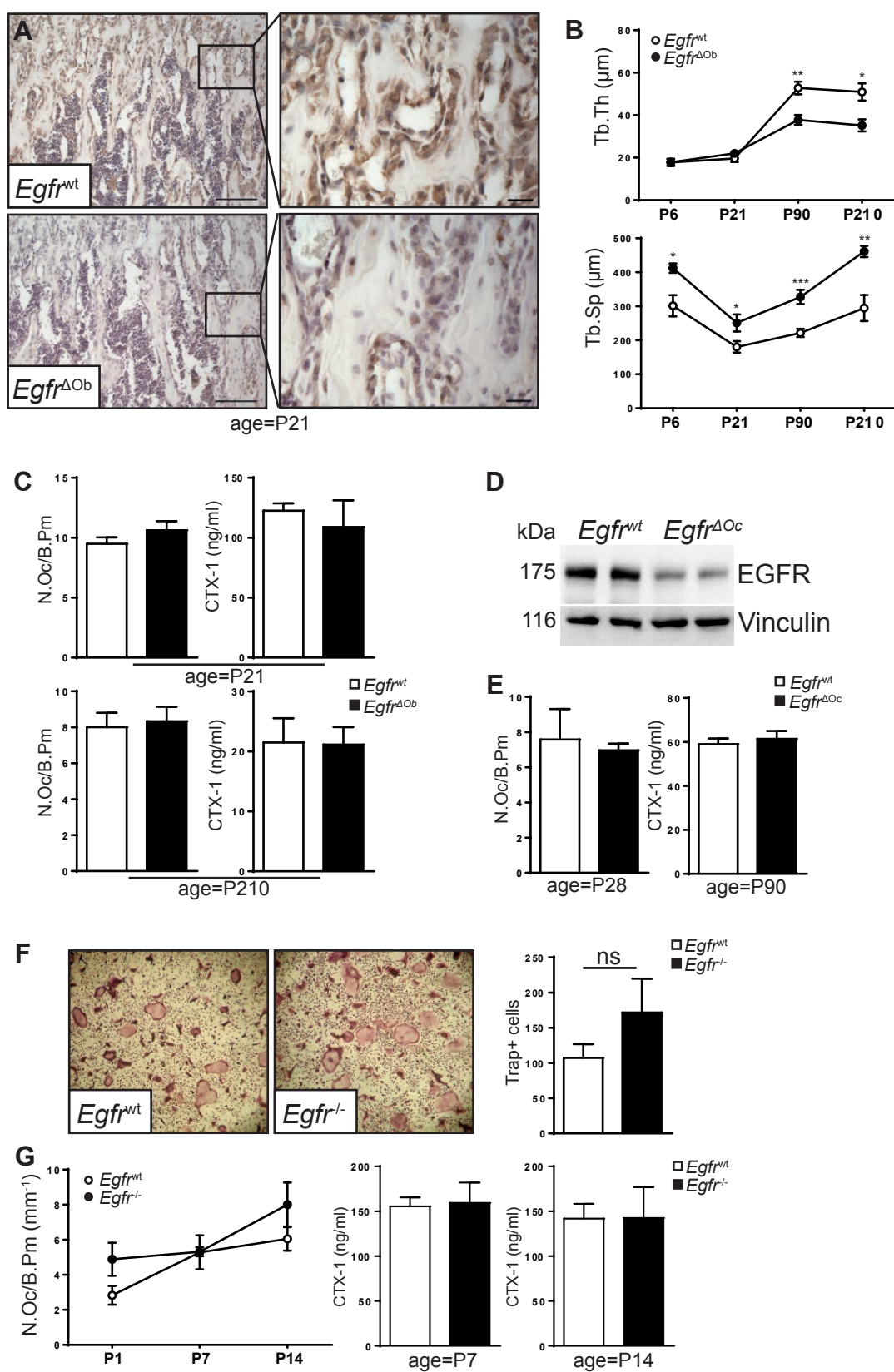

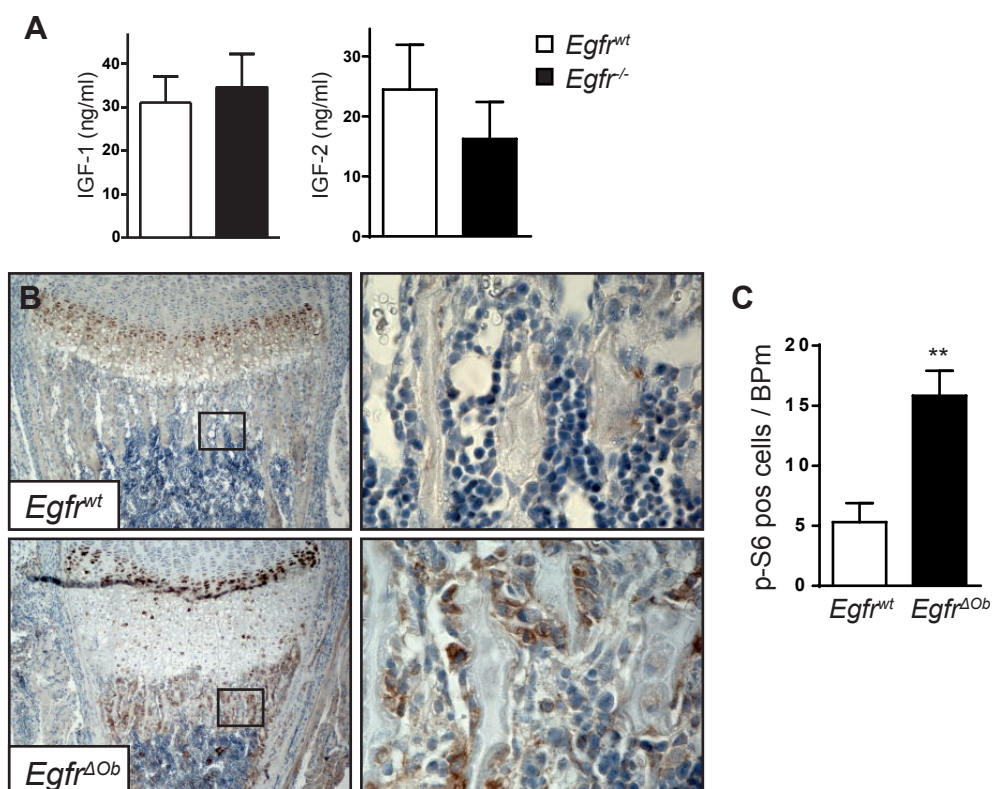

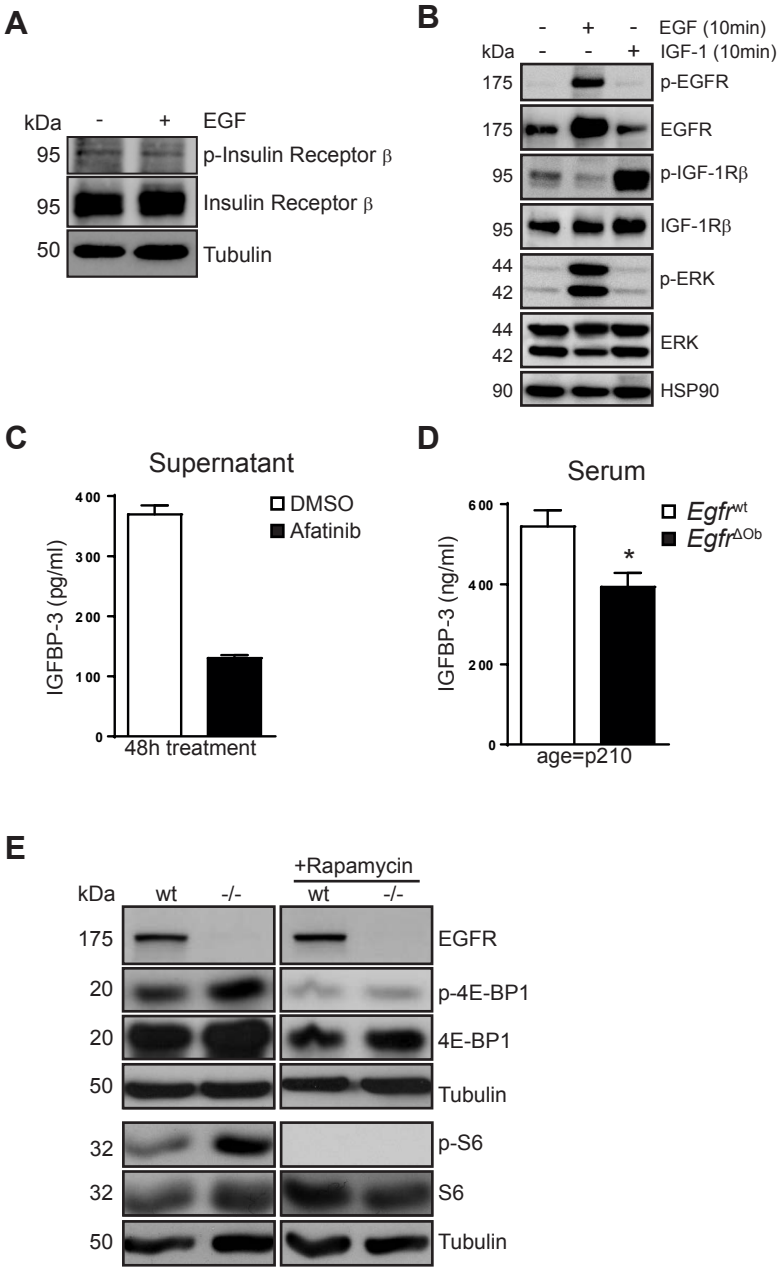

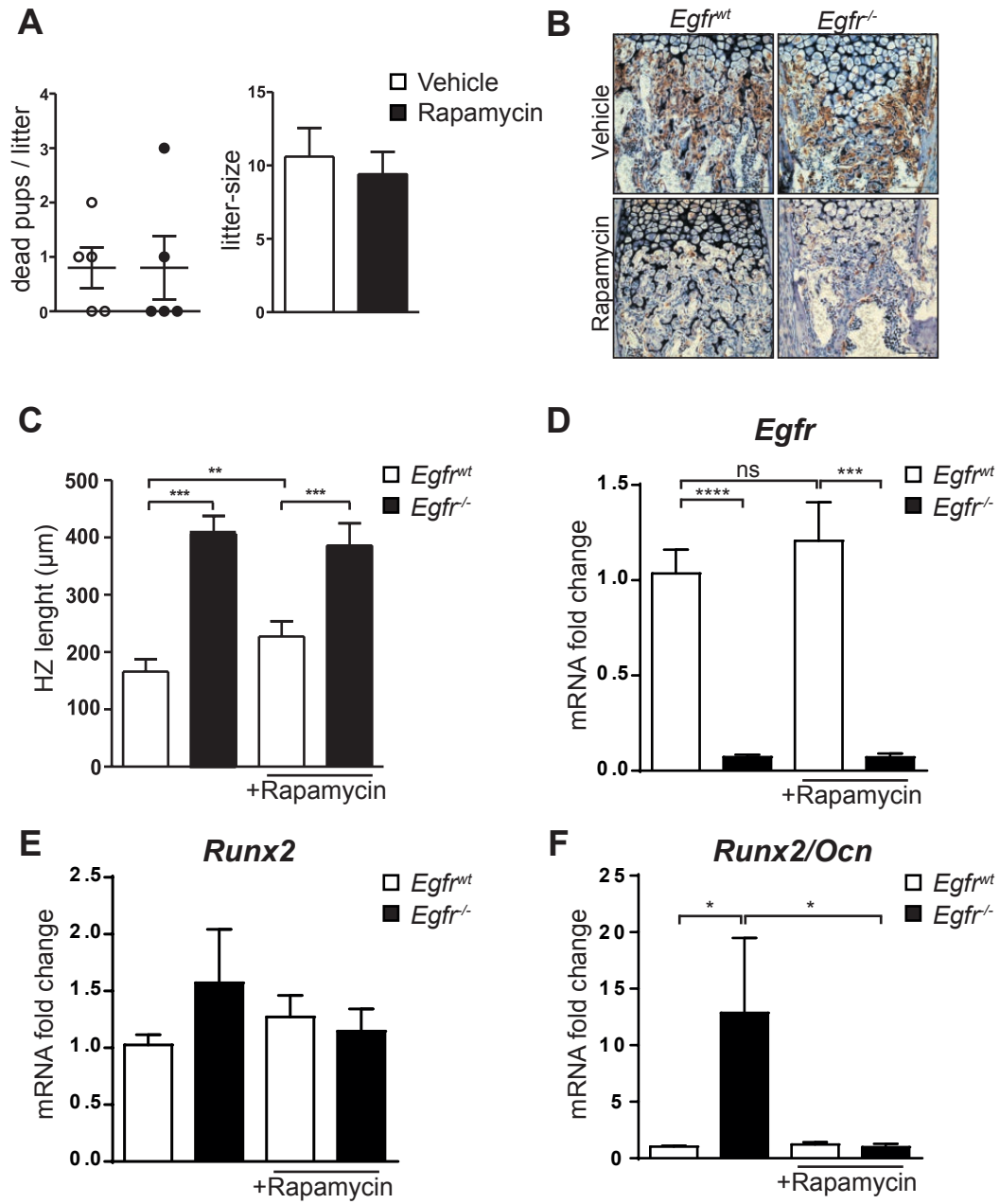

Supplement: Supplementary file 1 — Supplemental Figures S1-7 [file 41418_2017_54_MOESM1_ESM.pdf]
