# Supplementary material for: EGFR controls bone development by negatively regulating mTOR-signaling during osteoblast differentiation
Source: Cell Death Differ. 2018 Feb 14;25(6):1094–106. doi: 10.1038/s41418-017-0054-7 (PMC5988706; doi:10.1038/s41418-017-0054-7)
Supplement: Supplementary file 2 — Supplemental Table S1 [file 41418_2017_54_MOESM2_ESM.pdf]

Antibodies used for Western Blot

DIRECTED AGAINST	COMPANY	PRODUCT NO.	SOURCE
Actin	Sigma	A 2066	rabbit
p-Akt (Ser 473)	Cell signaling	4060	rabbit
p-Akt (Thr 308)	Cell signaling	9275	rabbit
Akt	Cell signaling	9272	rabbit
Cyclin D1	Santa Cruz	sc-450	mouse
p-4E-BP1 (Thr 37/46)	Cell signaling	9459	rabbit
p-4E-BP1 (Thr37/46) (236B4)	Cell signaling	2855	rabbit
4E-BP1	Cell signaling	9452	rabbit
4E-BP1	Cell signaling	9272	rabbit
EGFR (D38B1)	Cell signaling	4267	rabbit
EGFR	Upstate	06-129	Sheep
pERK1/2 (Thr202/Tyr204)	Cell signaling	9101	rabbit
ERK1	Santa Cruz	sc-93	rabbit
ERK2	Santa Cruz	sc-154	rabbit
HSP90	Cell signaling	4874	rabbit
p-IGF-1Rß (Tyr 1135/1136)/ IR (Tyr 1150/1151)	Cell signaling	3024	rabbit
IGF-1Rß	Cell signaling	3027	rabbit
IGFBP-3	R&D systems	AF775	goat
p-Insulin Receptor (Tyr1361)	abcam	ab60946	rabbit
Insulin Receptor (4B8)	Cell signaling	3025	rabbit
p-IRS-1/2 (Tyr 612 in humans = Tyr 608 in mice)	Santa Cruz	sc-17195-R	rabbit
p-IRS-1 (Ser 307)	Upstate	07-247	rabbit
p-IRS-1 (Ser 612)	Cell signaling	3193	mouse
IRS-1	Cell signaling	2382	rabbit
p-SAPK/JNK (Thr183/Tyr185)	Cell signaling	9255	mouse
SAPK/JNK	Cell signaling	9252	rabbit
p-mTOR (Ser 2448)	Cell signaling	2971	rabbit
p-mTOR (Ser2448)	Santa Cruz	sc-101738	rabbit
mTOR	Cell signaling	2972	rabbit
p-p70S6K	Cell signaling	9205	rabbit
p70S6K	Cell signaling	9202	rabbit
p-S6 (Ser 240/244)	Cell signaling	2215	rabbit
p-S6 (Ser 235/236)	Cell signaling	4856	rabbit
S6 Ribosomal Protein (54D2)	Cell signaling	2317	mouse
S6 Ribosomal Protein (5G10)	Cell signaling	2217	rabbit
α-Tubulin	Sigma	T9026	mouse
Vinculin	Sigma	V9131	mouse

Antibodies used for IHC

DIRECTED AGAINST	COMPANY	PRODUCT NO.	SOURCE
p-4E-BP1 (Thr37/46) (236B4)	Cell signaling	2855	rabbit
EGFR (D38B1)	Cell signaling	4267	rabbit
p-ERK (D13.14.4E)	Cell signaling	4370	rabbit
p-Histone H3 (Ser10)	Cell signaling	9701	rabbit
p-mTOR (Ser2448)	Santa Cruz	sc-101738	rabbit
Osteocalcin (Fl-100)	Santa Cruz	Sc-30044	rabbit
p-S6 (Ser235/236)	Cell signaling	4858	rabbit
PCNA (D3H8P)	Cell signaling	13110	rabbit
