# Supplementary material for: EGFR controls bone development by negatively regulating mTOR-signaling during osteoblast differentiation
Source: Cell Death Differ. 2018 Feb 14;25(6):1094–106. doi: 10.1038/s41418-017-0054-7 (PMC5988706; doi:10.1038/s41418-017-0054-7)
Supplement: Supplementary file 3 — Supplemental Figure and Table Legends [file 41418_2017_54_MOESM3_ESM.pdf]

Supplemental Figure S1. *Egfr*$^{-/-}$ mice show impaired mineralization. Related to Figure 1

(A) Skeletal preparations showing ribcages of 7-day-old WT and KO mice. **(B)** Movat staining of *Egfr*^{wt} and *Egfr*$^{-/-}$ tibiae at P14; scale: 100µm for both lower (left) and higher magnification (right).

Supplemental Figure S2. EGFR-deficient osteoblasts show reduced proliferation *in vitro.* Related to Figure 2. (A) Cumulative growth curve showing reduced cell number of *Egfr*$^{-/-}$ OBs; n=2 **(B)** Percentage of late apoptotic OBs as determined by FACS (Annexin V+, 7-AAD+); n=5 **(C)** BrdU incorporation in *Egfr*^{wt} and *Egfr*$^{-/-}$ OB cultures; n=4 **(D)** Western Blot analysis of WT and KO OBs on day 4 after isolation.

Supplemental Figure S3. EGFR is efficiently deleted in osteoblasts and long-bones of *Egfr*$^{\Delta Ob}$ mice. Related to Figure 3.

(A) Western blot analysis of protein lysates obtained from differentiated osteoblasts isolated from bone-marrow of mice with indicated genotypes. **(B)** EGFR IHC staining showing efficient deletion of EGFR in long bones of 6-day-old *Egfr*$^{f/f}$ Runx2-Cre mice. **(C)** qRT-PCR analysis of RNA isolated from cartilage or bone tissue of 2-day-old *Egfr*$^{f/f}$ and *Egfr*$^{\Delta Ob}$ littermates. Expression levels of osteoblastic (*Ocn*, *Opn*) and chondroblastic (*Col2a1*, *Col10a1*) genes were analyzed to confirm correct isolation of the corresponding tissue. **(D)** X-ray image of 3-months-old *Egfr*^{wt} and *Egfr*$^{\Delta Ob}$ littermate showing no differences in overall body length.

Supplemental Figure S4. EGFR deletion does not affect osteoclastogenesis. Related to Figure 4.

(A) Osteocalcin IHC staining showing reduced levels on the trabecular bone of a 21-days old *Egfr*$^{\Delta Ob}$ mouse; scales: 100µm (lower magnification) and 20µm (higher

magnification). **(B)** Histomorphometric analysis of WT/ΔOb long-bones at P6, P21, P90 and P210: Quantification of trabecular separation (Tb.Sp), and trabecular thickness (Tb.Th). P6: n=4 WT, 3 ΔOb. P21: n=5. P90: n=8 WT, 6 ΔOb. P210: n=4 WT, 6 ΔOb mice. **(C)** Osteoclast number on the trabecular bone of $Egfr^{wt}$ and $Egfr^{\Delta Ob}$ mice at P21 (n=5 WT, 7 ΔOb) and P210 (n=5 WT, 7 ΔOb) and CTX-1 as measured by ELISA in Serum at P21 (n=7 WT, 5 ΔOb) and P210 (n=5 WT, 7 ΔOb). **(D)** Western Blot analysis of bone-marrow derived osteoclasts isolated from $Egfr^{wt}$ and $Egfr^{f/f}$ LysM-Cre mice. **(E)** Osteoclast number on the trabecular bone at P28 (n=4) and CTX-1 serum levels at P90 (n=3 WT, 4 ΔOc) in $Egfr^{wt}$ and $Egfr^{f/f}$ LysM-Cre mice. **(F)** TRAP staining of differentiated osteoclasts isolated from $Egfr^{wt}$ and $Egfr^{-/-}$ mice; n=7. **(G)** Osteoclast number on the trabecular bone of $Egfr^{wt}$ and $Egfr^{-/-}$ mice. P1: n=6. P7: n=8 WT, 7KO. P14: n=6 WT, 5 KO mice. CTX-1 serum levels at P7 (n=10 WT, 7 KO) and P14 (n=4 WT, 5 KO).

Supplemental Figure S5. EGFR deletion does not affect IGF-1 and -2 levels *in vitro*. Related to Figure 5.

(A) IGF-1 and IGF-2 protein levels in the supernatant of osteoblasts after 14 days of differentiation. IGF-1: n=4. IGF-2: n=3 WT, 4 KO. **(B)** IHC staining against p-S6 Protein in long-bones of 7-day-old $Egfr^{wt}$ and $Egfr^{\Delta Ob}$ littermates. **(C)** Quantification of p-S6 staining; n=5 WT, 6 ΔOb.

Supplemental Figure S6. Rapamycin downregulates EGFR dependent hyper-activation of 4E-BP1 and S6 in osteoblasts. Related to Figure 6.

(A) Western blot analysis of differentiated WT osteoblasts (D21) untreated or cultured with EGF (100ng). **(B)** Western Blot analysis of starved (24h, 0%FCS) differentiated WT osteoblasts (D21) after EGF (20ng/ml) or IGF-1 (100ng/ml) stimulation for 10

minutes. **(C)** IGFBP-3 Elisa of supernatant from starved osteoblast precursor cells after 48h DMSO or Afatinib (1µM) treatment (n=3). **(D)** IGFBP-3 Elisa of serum from 7-months old *Egfr*^{wt} and *Egfr*$^{\Delta Ob}$ mice (n=6). **(E)** Western Blot analysis of protein lysates obtained from differentiated primary osteoblasts cultured for 21 days with vehicle (DMSO) or rapamycin (10nM).

Supplemental Figure S7. Rapamycin treatment prevents mTOR pathway activation in utero. Related to Figure 7.

(A) Dead pups per litter and litter-size on E18.5 after Rapamycin or Vehicle treatment **(B)** IHC staining showing phosphorylated S6 protein expression in distal femur sections of Rapamycin/vehicle treated *Egfr*^{wt} and *Egfr*$^{-/-}$ embryos on E18.5. **(C)** Quantification of length of hypertrophic chondrocyte zone measured in distal femural growth plates of Rapamycin/Vehicle treated embryos. n=6 WT, 7 KO mice for vehicle and 6 WT, 7 KO mice for rapamycin treatment. **(D,E,F)** mRNA expression levels of **(D)** *Egfr* (n=5 WT, 5 KO mice for vehicle and 3 WT, 3 KO mice for rapamycin treatment) **(E)** *Runx*2 and **(F)** *Runx2/Ocn* (n=9 WT, 5 KO mice for vehicle and 7 WT, 5 KO mice for rapamycin treatment) as measured by qRT-PCR from RNA isolated from embryonic femurs at E18.5.

Supplemental Table S1: Antibodies used for WB and IHC
